# A Giant Magneto‐Superelasticity of 5% Enabled by Introducing Ordered Dislocations in Ni_34_Co_8_Cu_8_Mn_36_Ga_14_ Single Crystal

**DOI:** 10.1002/advs.202401234

**Published:** 2024-04-24

**Authors:** Qijia Yu, Jingmin Wang, Chuanxin Liang, Jiaxi Meng, Jinyue Xu, Yang Liu, Shiteng Zhao, Xuekui Xi, Chuanying Xi, Ming Yang, Chen Si, Yangkun He, Dong Wang, Chengbao Jiang

**Affiliations:** ^1^ School of Materials Science and Engineering Key Laboratory of Advanced Aerospace Materials and Performance (Ministry of Education) Beihang University Beijing 100191 P. R. China; ^2^ Center of Microstructure Science Frontier Institute of Science and Technology State Key Laboratory for Mechanical Behavior of Materials Xi'an Jiaotong University Xi'an Shaanxi 710049 P. R. China; ^3^ Beijing National Laboratory for Condensed Matter Physics Institute of Physics Chinese Academy of Sciences Beijing 100190 P. R. China; ^4^ Anhui Province Key Laboratory of Condensed Matter Physics at Extreme Conditions High Magnetic Field Laboratory of the Chinese Academy of Science Hefei Anhui 230031 P. R. China; ^5^ National High Magnetic Field Center and School of Physics Huazhong University of Science and Technology Wuhan 430074 P. R. China

**Keywords:** magneto‐superelasticity, ordered dislocations, strain

## Abstract

Elasticity, featured by a recoverable strain, refers to the ability that materials can return to their original shapes after deformation. Typically, the elastic strains of most metals are well‐known 0.2%. In shape memory alloys and high entropy alloys, the elastic strains can be several percent, as called superelasticity, which are all triggered by external stresses. A superelasticity induced by magnetic field, termed as magneto‐superelasticity, is extremely important for contactless work of materials and for developing brand‐new large stroke actuators and high efficiency energy transducers. In magnetic shape memory alloys, the twin boundary motion driven by magnetic field can output a strain of several percent. However, this strain is unrecoverable when removing the magnetic field and hence it is not magneto‐superelasticity. Here, a giant magneto‐superelasticity of 5% in a Ni_34_Co_8_Cu_8_Mn_36_Ga_14_ single crystal is reported by introducing arrays of ordered dislocations to form preferentially oriented martensitic variants during the magnetically induced reverse martensitic transformation. This work provides an opportunity to achieve high performance in functional materials by defect engineering.

## Graphical abstract

Ordered dislocations are introduced into a Ni_34_Cu_8_Co_8_Mn_36_Ga_14_ single crystal to effectively control the preferential orientation of martensitic variants during cycles of magnetic field induced reverse martensitic transformation. A giant magneto‐superelasticity of 5% is realized. This work provides an opportunity to develop advanced superelastic materials and related high performance devices.

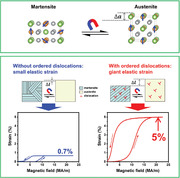

## 1 Introduction

Modern engineering and medical techniques, for example stroke controlling, transducer actuating, precision machining, energy harvesting, etc, require high‐performance devices of actuators and energy transducers. If a material can output recoverable elastic strain under a magnetic field, it will be benefit for contactless work of materials in high performance devices. Ferromagnetic materials generally output recoverable elastic strain, termed as magnetostriction and also magneto‐elasticity, when they are applied a magnetic field. Usually, TbDyFe alloys possess the highest magnetostriction which is within 0.2%.^[^ ^1^, ^2^ ^]^ Recoverable magneto‐elastic strains within 0.6% have been reported in NiCoMnX (X = In, Sn, Sb, Al, Ga) magnetic shape memory materials.^[^ ^3^, ^4^, ^5^, ^6^, ^7^, ^8^, ^9^, ^10^, ^11^, ^12^, ^13^, ^14^, ^15^ ^]^ A giant magneto‐elasticity is expected all the time for developing high performance devices.

Recoverable elastic strain of 0.2% exists for most metals.^[^ ^16^ ^]^ Large recoverable elastic strains of serval percent, as called superelasticity, have been observed in some metallic materials such as shape memory alloys,^[^ ^17^, ^18^, ^19^, ^20^, ^21^, ^22^, ^23^, ^24^, ^25^, ^26^, ^27^, ^28^, ^29^ ^]^ high entropy alloys,^[^ ^30^, ^31^, ^32^ ^]^ metallic glasses,^[^ ^33^, ^34^, ^35^ ^]^ gum‐metal,^[^ ^37^, ^38^, ^39^ ^]^ metallic nanocomposites,^[^ ^40^, ^41^ ^]^ etc. All these superelasticities are obtained under external stress, which can not be utilized for contactless work of materials in the devices.

Several percent of strain has been obtained in NiMnGa magnetic shape memory alloys through magnetic field‐induced twin boundary motion.^[^ ^42^, ^43^, ^44^, ^45^, ^46^, ^47^, ^48^ ^]^ For example the strain of 5%, 9.5% and 12% has been achieved in in Ni_48_Mn_31_Ga_21_,^[^ ^49^ ^]^ Ni_48.8_Mn_29.7_Ga_21.5_^[^ ^50^ ^]^ and Ni_46_Mn_24_Ga_22_Co_4_Cu_4_^[^ ^51^ ^]^ alloys, respectively. However, the strain is unrecoverable with very small output stress of 5 MPa. Strain of 3% has been reported in a NiCoMnIn single crystal due to magnetic field‐induced reverse martensitic transformation (MFIRMT). Unfortunately, this strain is also unrecoverable, as called magnetically controlled one‐way shape memory effect.^[^ ^52^ ^]^ The magnetically controlled one‐way shape memory effect of the NiCoMnIn single crystal is also observed in a pulsed magnetic field.^[^ ^53^ ^]^ This strain is recovered by applying a large compressive stress of 125 MPa, which require a complicate system for applications.^[^ ^54^ ^]^ Moreover, in this case, the magnetic field stabilizes the high temperature ferromagnetic austenite phase but the compressive stress contrarily stabilizes the low temperature martensite phase. This principally limits the achievement of higher strain. Nowadays, in a compressive stress‐free state (free‐standing), less than 0.6% recoverable magneto‐elastic strains can be obtained.^[^ ^12^ ^]^ Until now, a magneto‐superelasticity with several percent of recoverable strain simply triggered by magnetic field has never been reported in the free‐standing state.

**Figure 1:**
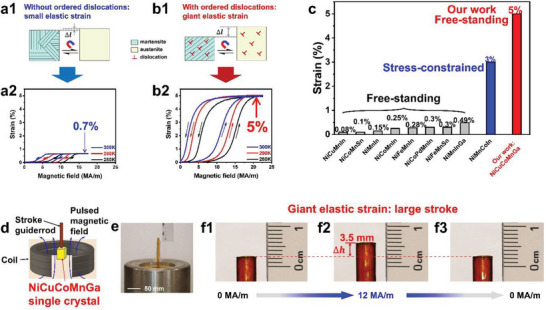
Magneto‐elasticity of the Ni_34_Co_8_Cu_8_Mn_36_Ga_14_ single crystal. a1) and b1) Schematic illustration of self‐accommodated and preferentially oriented martensitic variants without and with ordered dislocations. a2) and b2) The corresponding experimental results on the small and giant magneto‐elastic strain, respectively. c) Comparison between our result and the previous reports.^[^ ^6^, ^7^, ^9^, ^10^, ^11^, ^12^, ^13^, ^15^, ^49^ ^]^ d) Schematic illustration of a device designed based on the Ni_34_Co_8_Cu_8_Mn_36_Ga_14_ single crystal to test the stroke in case of the giant magneto‐superelasticity. e) Real photo of the device. f1) – f3). At room temperature, large stroke of the device is observed in case of the giant magneto‐superelasticity. A movie corresponding to f) is recorded (Movie S1, Supporting Information).

Herein, our idea is to introduce ordered dislocations into the free‐standing samples to control the formation of preferential orientation of martensite variants. A giant magneto‐superelasticity during the MFIRMT is expected macroscopically, as schematically shown in **Figure** **1**a1,b1. Single crystal of Ni_34_Co_8_Cu_8_Mn_36_Ga_14_ alloy has been grown, which possesses the MFIRMT around room temperature as reported in our previous work.^[^ ^55^ ^]^ A training of stress constrained transformation cycles is proposed, which is always applied for realization of two‐way shape memory effect for guiding the orientation of martensite variants.^[^ ^56^, ^57^, ^58^ ^]^ Ordered dislocations with the Burgers vector [110] are introduced into the free‐standing single crystal during the constrained transformation cycles. Preferential orientation of the martensitic variants is formed during the reversible MFIRMT by the local internal stress generated by the ordered dislocations. A giant magneto‐superelasticity of 5% is achieved in the free‐standing Ni_34_Co_8_Cu_8_Mn_36_Ga_14_ single crystal with the output stress of 100 MPa. This work provides an opportunity to achieve high performance in functional materials by defect engineering.

## 2 Results and Discussions

### 2.1 Giant Magneto‐Superelasticity of the Ni_34_Co_8_Cu_8_Mn_36_Ga_14_ Single Crystal

Based on the phase structure and orientation relationship in the Ni_34_Co_8_Cu_8_Mn_36_Ga_14_ single crystal (Figures S1 and S2, Supporting Information), we measured the elastic strain along the 〈001〉_*A*_ direction (A denotes the austenite phase). The measurements were carried out at a steady high magnetic field facility (SHMFF). For the as‐grown single crystal, the elastic strain detected under the magnetic field is only 0.7% (Figure 1a2). We performed a stress‐constrained transition cycling (SCTC) training for the single crystal by applying a compressive stress of 40 MPa (details described in the methods). After the SCTC training, the applied external stress was removed. In the free‐standing SCTC‐treated single crystal, the magneto‐superelasticity with the recoverable strain up to 5% is detected at 280 K, 290 K, and 300 K, as shown in Figure 1b2. Moreover, the recoverable strain of 5% is still output when the Ni_34_Co_8_Cu_8_Mn_36_Ga_14_ single crystal is applied compressive stress of 100 MPa (Figure S3, Supporting Information). This indicates the ability of outputting high stress of the giant magneto‐superelasticity. Compared with the recoverable strain produced by the MFIRMT in the free‐standing or stress‐constrained alloys, the 5% recoverable strain is the largest as shown in Figure 1c.

A device driven by a pulsed magnetic field is designed based on the Ni_34_Co_8_Cu_8_Mn_36_Ga_14_ single crystal, as shown in Figure 1d,e. The pulse width of the magnetic field is 10 ms. At room temperature large stroke is resulted from the giant magneto‐superelasticity of the SCTC‐treated single crystal, as shown in Figure 1f. The corresponding movies are included in the supplementary materials (Movies S1 and S2). From the viewpoint of potential applications, the delayed response of the Ni_34_Co_8_Cu_8_Mn_36_Ga_14_ single crystal to the pulsed magnetic field is an important parameter. *M*‐*H* curves of the Ni_34_Co_8_Cu_8_Mn_36_Ga_14_ single crystal are measured in a pulsed magnetic field with the pulse width of 8 ms (Figure S4, Supporting Information). The lagged response is ≈0.1 ms, indicating quick response of the Ni_34_Co_8_Cu_8_Mn_36_Ga_14_ single crystal to the magnetic field.

### 2.2 Magnetic Field‐Induced Reverse Martensitic Transformation

**Figure 2:**
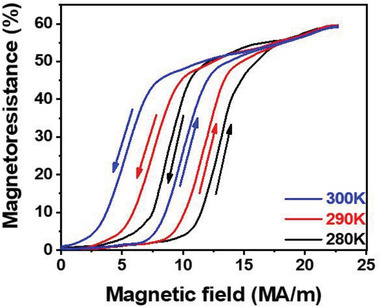
Magnetic field‐induced reverse martensitic transformation in the Ni_34_Co_8_Cu_8_Mn_36_Ga_14_ single crystal. Dependence of the magnetoresistance on the magnetic field is measured at 280 K, 290 K, and 300 K, respectively. The abrupt change of the magnetoresistance and the obvious hysteresis, together with the structural analysis (Figure S1, Supporting Information), give evidence of the reversible reverse martensitic transformation induced by the magnetic field.

Temperature dependence of X‐ray diffractions confirm that the Ni_34_Co_8_Cu_8_Mn_36_Ga_14_ alloy undergoes a martensitic transition between face‐centered cubic (fcc) austenite phase and the body‐centered tetragonal (bct) martensite phase at ≈320 K (Figure S2a, Supporting Information). Quite large lattice distortion and abrupt change of the electrical resistance are observed during the martensitic transformation (Figure S2b,c, Supporting Information). Magnetism measurements and density functional theory (DFT) calculations further prove that the austenite phase is ferromagnetic and the martensite phase is in spin glass state (Figure S5 and Tables S1 and S2, Supporting Information). Therefore, the Ni_34_Co_8_Cu_8_Mn_36_Ga_14_ alloy undergoes a metamagnetic martensitic transformation between the spin glass martensite phase and the ferromagnetic austenite phase. Due to the large difference in magnetization, i.e., Δ*M*, between both phases, applying magnetic field will result in a difference in the Zeeman energy μ_0_ *H* · Δ*M*. From the viewpoint of thermodynamics, the Zeeman energy can supply additional energy to the difference in the Gibbs free energy required to drive the reverse martensitic transformation. According to this analysis, the reverse martensitic transformation is expected to be induced simply by applying magnetic field. This was experimentally confirmed by measuring the field dependence of electrical resistance, as shown in **Figure** **2**.

The measurement was performed with the initial state of the martensite phase. A significant change in the electrical resistance, up to 50%, is observed. Generally, such a large change in electrical resistance only results from a first‐order structural transition. Therefore, electrical resistance measurements further confirm the magnetic field‐induced phase transformation. Except the above measurement in the steady magnetic field, field dependence of the magnetization is also measured in a steady magnetic field and a pulsed magnetic field (Figures S4 and S6, Supporting Information). It is confirmed that the phase transition is also driven by the pulsed magnetic field, indicating the fast response of the phase transition to the magnetic field.

### 2.3 Ordered Dislocations Introduced by SCTC Training

**Figure 3:**
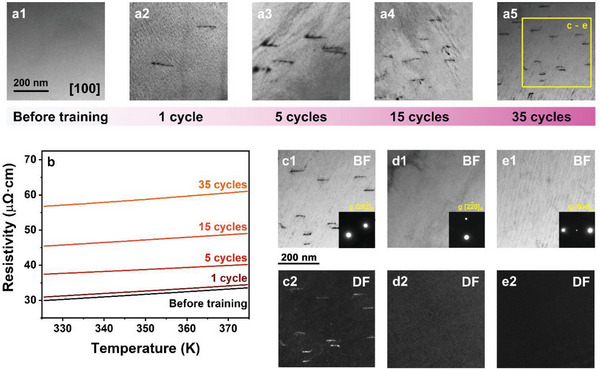
Ordered dislocations introduced in to the Ni_34_Cu_8_Co_8_Mn_36_Ga_14_ single crystal by the SCTC training. The TEM images a1–a5) and electronic resistivity dependent curves b) of the Ni_34_Cu_8_Co_8_Mn_36_Ga_14_ single crystal before and after various cycles of SCTC training. Two‐beam bright‐field images c1), d1), e1) and weak beam dark‐field images c2), d2), e2) with *g* vectors of ${\left[20\bar{2}\right]}_{A}$, ${\left[2\bar{2}0\right]}_{A}$ and [004]_*A*_ of Ni_34_Cu_8_Co_8_Mn_36_Ga_14_ single crystal after 35 cycles of SCTC training.

**Figure** **3**a1–a5 shows the TEM images of untreated Ni_34_Co_8_Cu_8_Mn_36_Ga_14_ single crystal and the single crystals treated after different SCTC cycles. Compared with the untreated single crystal dislocations are introduced by the SCTC training. With the increased SCTC cycles density of the dislocations is obviously increased, which is also verified by measurements of electrical resistivity for the high temperature austenite, as shown in Figure 3b. Compared with the untreated single crystal, the electrical resistivity is increased after the SCTC training. According to Matthiessen's rule, the increased electrical resistivity is attributed to increased density of dislocations introduced by the SCTC training. TEM analysis is performed to determine the Burgers vectors of the dislocations. For the convenient analysis, the high temperature austenite phase is analyzed. The austenite phase possesses fcc structure. Theoretically there are mainly six Burgers vectors parallel to 〈110〉_*A*_ directions. According to “***b*** · ***g*** = 0 invisibility” criterion, where ***b*** and ***g*** are Burgers vector of dislocations and operation vector of diffractions, it is necessary to obtain the information of electronic diffractions along 〈220〉_*A*_ and 〈004〉_*A*_ directions to determine the Burgers vectors of the dislocations. Figure 3c1‐e2 are bright‐field and dark‐field images with ***g*** vectors of ${\left[20\bar{2}\right]}_{A}$, ${\left[2\bar{2}0\right]}_{A}$, and [004]_*A*_ addressed by using TEM on the (100)_*A*_ plane of the single crystal. The result of ***b*** · ***g*** = 0 for ${\left[2\bar{2}0\right]}_{A}$ and [004]_*A*_ suggests that all the dislocations have the same Burgers vector of [110]_*A*_. So it is confirmed that ordered dislocations are introduced into the Ni_34_Co_8_Cu_8_Mn_36_Ga_14_ single crystal by SCTC training.

### 2.4 The Preferentially Oriented Martensitic Variants

The quite large lattice distortion accompanying the martensitic transformation of the Ni_34_Co_8_Cu_8_Mn_36_Ga_14_ single crystal gives rise to the potential for a giant magneto‐superelasticity. Usually self‐accommodated martensitic variants, i.e., randomly oriented variants, form during the martensitic transformation. The microscopically large lattice distortion strain is averaged macroscopically by the randomly oriented variants, restricting output of the magneto‐superelasticity.

**Figure 4:**
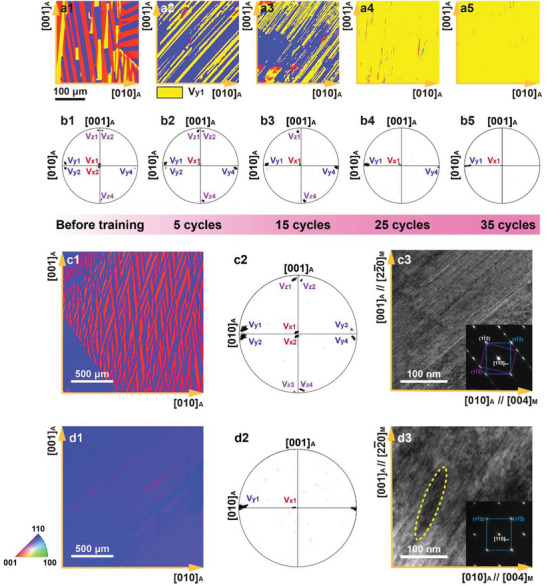
Preferentially oriented martensitic variants of the Ni_34_Co_8_Cu_8_Mn_36_Ga_14_ single crystal. The martensitic variants are characterized by using EBSD and TEM addressed on the (100)_*A*_ plane of the Ni_34_Co_8_Cu_8_Mn_36_Ga_14_ single crystal. a1–a5) and b1–b5) Evolution of the orientation of the martensitic variants with the cycle numbers of the SCTC training as characterized by EBSD images and corresponding pole figures, respectively. c,d) Detailed analysis on the as‐grown and SCTC‐treated single crystals. c1) and d1) EBSD graphs for the variant with the large visual field. c2) and d2) Pole figures for the variant orientations originated from c1) and d1). c3,d3) TEM graphs and SAED patterns (insets) of the twinning microstructure.

We performed SCTC training for the Ni_34_Co_8_Cu_8_Mn_36_Ga_14_ single crystal and a giant magneto‐superelasticity of 5% is realized. In comparison, strain of only 0.7% is observed for the as‐grown single crystal without SCTC training. The preferentially oriented martensitic variants are believed to form during the martensitic transformation for the output of the giant magneto‐superelasticity. The effect of SCTC training cycles on the preferentially oriented martensitic variants is investigated by electron back scattering diffraction (EBSD) images and the related pole figures, as shown in **Figure** **4**a1–a5 and Figure 4b1–b5, respectively. In the untreated single crystal, configuration of self‐accommodated martensitic variants composing of randomly oriented variants is observed, as shown in Figure 4a1,b1. With the increase of SCTC training cycles, preferentially oriented martensitic variants gradually form.

Figure 4c,d gives a comparison in the configuration of the martensitic variants of the untreated single crystal and after 35 cycles of the SCTC training. Compared with the as grown single crystal, only two variants, V_y1_ and V_x1_, are observed after the SCTC training. The volume fraction of the predominant variant V_y1_ is 98.7%, and that of variant V_x1_ is only 1.3%. Preferentially oriented martensitic variants form. According to selected area electron diffraction (SAED, Figure S2, Supporting Information), the crystallographic orientation relationship between the austenite phase and the martensitic variant V_y1_ is ${\left(1\bar{1}1\right)}_{A}\;//\;{\left(101\right)}_{M}$, ${\left[110\right]}_{A}\;//\;{\left[11\bar{1}\right]}_{M}$. When the martensitic variant V_y1_ transforms into the austenite phase, the bct lattice of the martensite phase is elongated along the [110]_*M*_ and ${\left[1\bar{1}0\right]}_{M}$ directions and is shortened along the [001]_*M*_ direction, which corresponds to the [100]_*A*_, [001]_*A*_, and [010]_*A*_ directions in the austenite coordinate system. Considering the 6.2° tilt of the tetragonal lattice during shear along the ${\left[110\right]}_{A}\;//\;{\left[11\bar{1}\right]}_{M}$ direction, the elongation along the [100]_*A*_ direction is the largest as 7.7% calculated from the lattice parameters *a* = *b* = *c* = 0.5859 nm of the austenite phase and *a* = *b* = 0.3847 nm and *c* = 0.6734 nm of the martensite phase at 320 K as shown (Figure S1, Supporting Information). At the same time, micro‐twin structure is observed in the variants, which also affect the output of the superelasticity (Figure 4c3). After the SCTC training, detwinning occurs (Figure 4d3). Therefore, in the case of achieving preferentially oriented martensitic variants via SCTC training, the microscopic large lattice distortion strain can macroscopically output the giant magneto‐superelasticity along the [100]_*A*_ direction of the single crystal.

### 2.5 Role of the Ordered Dislocations in the Formation of Preferentially Oriented Martensitic Variants

**Figure 5:**
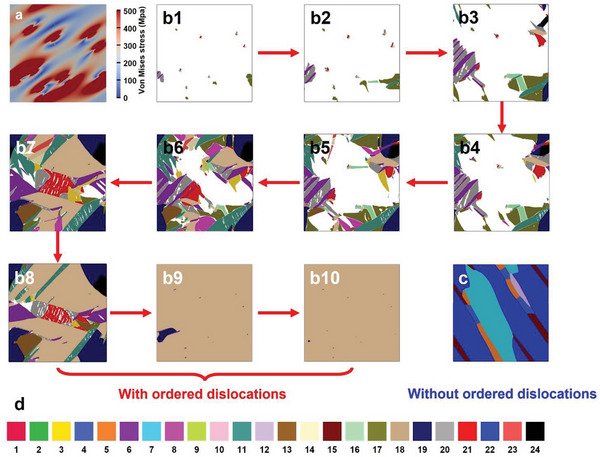
Phase field simulations. a) Distribution of local internal stress simulated based on the bright‐field image in Figure 3c1). b) Simulated microstructure evolution during the transition from austenite to martensite of the SCTC‐treated Ni_34_Co_8_Cu_8_Mn_36_Ga_14_ single crystal. Variant 18 is selected by introducing the ordered arrays of dislocations, as shown in b10). c) Random choice of variants in the single crystal without ordered dislocations. d) 1 – 24 represent the martensitic variants with different orientations.

In order to reveal the role of the ordered dislocations in the formation of the preferentially oriented martensitic variants after SCTC training, 2D phase field simulations are performed for themartensitic variants to reveal the role of the dislocations. A 400 nm × 400 nm square system is built according to Figure 3c1,c2, from which the local internal stress field caused by the dislocations is analytically calculated, and the distribution of the von Mises stress is shown in **Figure** **5**a. The dislocations distort the lattice and generate a local internal stress field. A phase field model considering the contribution of the internal local stress to the total energy of the phase transition system was utilized for the simulation, and the stochastic time‐dependent Ginsburg–Landau equation was used as the governing equation (details in the Supporting Information).

The simulated microstructure evolution during the transformation from the austenite phase to the martensite phase is shown in Figure 5b for the SCTC‐treated single crystal. Theoretically, 24 variants can form, as shown in Figure 5d, which are numbered 1, 2, 3, etc, and are represented by different colors. At the initial stage of the phase transition, the martensitic domains nucleate around the dislocations due to the large local internal stress field with demagnetization. Then, the martensitic domains grow and contact other domains (Figure 5b2–b6). Variant 18 shows an advantage in competition, and the interfaces between variant 18 and the other variants move toward the other variants, leading to a growth of variant 18 and shrinkage till disappearance of the other variants (Figure 5b7–b10). The [110]_*A*_ Burgers vector of the dislocations is parallel to the shear direction corresponding to variant 18. Therefore, variant 18 has the lowest energy in terms of the local stress fields caused by the screw dislocations (Figure 5a). For comparison, in case of without the ordered dislocations, the simulated martensite microstructure is composed of randomly oriented variants (Figure 5c). Therefore, the ordered dislocations are confirmed to be the key factor in the selection of the martensitic variants, which is important for the output of the giant magneto‐superelasticity.

## 3 Conclusions

A giant magneto‐superelasticity of 5% is achieved in the Ni_34_Co_8_Cu_8_Mn_36_Ga_14_ single crystal. The proposed idea of controlling preferentially orientation of martensite variants by introducing ordered dislocations is verified. The training of stress constrained transformation cycles inducing the ordered dislocations is realizable. Phase field simulations classify the effect of the ordered dislocations on the formation of preferentially oriented martensitic variants with the giant magneto‐superelasticity. Our work provides an attractive strategy to access high performance functional materials by defect engineering.

## 4 Experimental Section

### Material Preparations

The Ni_34_Co_8_Cu_8_Mn_36_Ga_14_ alloy was prepared by arc melting with high‐purity Ni, Co, Cu, Mn, and Ga elements. An additional 3% Mn was added to compensate for the vaporization of Mn during melting. Single crystals were grown by using the light heating zone melting method under an Ar atmosphere. The orientation of single crystals was determined by back‐reflection Laue X‐ray diffraction. Rectangular samples of 4 mm × 4 mm × 5 mm were cut along the [100]_*A*_, [010]_*A*_ and [001]_*A*_ directions (A denotes the austenite phase) of the single crystal for superelasticity measurements.

### Stress‐Constrained Transition Cycling (SCTC) Training

A simple device was designed by using non‐magnetic BeCu alloys (details in the supplementary information). A compressive stress of 40 MPa was supplied on the Ni_34_Co_8_Cu_8_Mn_36_Ga_14_ single crystal by BeCu disc springs. The SCTC training was performed for the stress‐constrained single crystal by heating/cooling cycles between 573 K and 77 K, which covered the martensitic transformation temperature range of the Ni_34_Co_8_Cu_8_Mn_36_Ga_14_ alloy. The single crystal was SCTC treated for different cycles. Before training, the single crystals were annealed at 773 K for 6 h and furnace cooled to eliminate residual stress. After the SCTC training, the applied compressive stress of 40 MPa was removed. Then, the single crystal was in a free‐standing state during the superelasticity measurements.

### Phase Transition Analysis

The thermally induced phase transition was analyzed by measuring the temperature‐dependent electrical resistance between 200 K and 400 K with a heating/cooling rate of 5 K min^−1^. The magnetic field‐induced phase transition was analyzed by measuring the magnetic field‐dependent magnetization and electrical resistance at the steady high magnetic field facility (SHMFF) located in the High Magnetic Field Laboratory of the Chinese Academy of Science. The magnetic field strength was up to 22.5 MA m^−1^. The increase speed of the magnetic field was 80 kA m^−1^s^−1^. The measurements were performed at 280 K, 290 K, 300 K, and 310 K. The magnetic field‐induced phase transformation was also analyzed by magnetization measurements at 300 K on the pulse high magnetic field facility (PHMFF) located at Wuhan National High Magnetic Field Center. The magnetic field strength was up to 32.5 MA m^−1^. The pulse width was 8 ms.

### Microstructure Analysis

The orientation of martensite variants was analyzed by electron backscatter diffraction (EBSD). The samples were vibration polished to eliminate the surface stress. In situ X‐ray diffraction (XRD) was performed upon heating from 240 to 480 K to confirm the crystal structure evolution during the phase transition. Transmission electron microscope (TEM) was adopted to characterize the microscopic structures. Samples for TEM analysis were prepared by the ion milling method. Characterization of the twin microstructure was performed in the martensite state. Characterization of dislocations were performed in the austenite phase. The two‐beam and weak beam methods were used to take bright‐field and dark‐field images to determine the Burgers vectors of dislocations.

### Magnetism Analysis

The magnetism of the martensite phase was analyzed by measuring the FC/ZFC temperature‐dependent magnetization (*M*‐*T*) and AC susceptibility (*χ*‐*T*) on a magnetic properties material system (MPMS). The temperature range was 10 K – 350 K. The magnetic field strength used for the *M*‐*T* measurement was 500 Oe. *χ*‐*T* curves were measured at frequencies of 5 Hz, 19 Hz, 188 Hz, and 1000 Hz and with an amplitude of 3 Oe. Limited by the highest temperature (320 K) available at the SHMFF, *M*‐*H* curves at 360 K (austenite phase) and 260 K (martensite phase) were measured on a physical property measurement system (PPMS) equipped with a superconductor magnet supplying magnetic field of 11.2 MA m^−1^.

### Measurement of the Electrical Resistivity

The electrical resistivity was measured by PPMS equipped with electrical transport. The single crystals after 0, 1, 5, 15, 35 cycles of SCTC training were cut into pieces of 4 mm × 2 mm × 0.5 mm. The temperature range was 300 K – 400 K. All the samples are heated to 500 K to remain single austenite phase state. The direction of 4 mm is measured direction by four‐wire method and along the [001] of austenite single crystal.

### Measurement of the Magneto‐Superelasticity

The magneto‐superelasticity was measured by using special strain gauges (KFGS‐02‐120‐C1‐11, Kyowo Company) with a strain range of 5%. The strain gauges were adhered to the single crystals with glue (CC‐33A), which could be subjected to temperatures ranging from 77 to 400 K. We adhered a strain gauge to a glass plate and by applying magnetic field it was confirmed that the magnetic field has no interference on the signal of the strain gauge. The magneto‐superelasticity measurement was performed at the SHMFF under the same conditions as mentioned above. At the same time, another free‐standing strain gauge was also used to obtain measurements to eliminate the influence of the conditions. The magneto‐superelasticity was measured at 280 K, 290 K, 300 K, and 310 K. For each measurement, the single crystal was initially cooled to 250 K and then heated to the temperature selected for magneto‐superelasticity measurement. A device for stroke measurements was built with the Ni_34_Co_8_Cu_8_Mn_36_Ga_14_ single crystals located in a pulsed magnetic field with the field up to 12 MA m^−1^ and the pulse width of 12 ms. A glass rod acting as the stroke guide rod was put on the single crystals. Movies were recorded by a camera during the actions of the device.

### DFT Calculations

Our spin‐polarized DFT calculations were performed using the projector augmented wave method in the DFT framework as implemented in the Vienna ab initio simulation package.^[^ ^59^ ^]^ The generalized gradient approximation of Perdew‐Burke‐Ernzerhof was adopted for the exchange‐correlation functional.^[^ ^60^, ^61^ ^]^ The kinetic energy cutoff for the plane‐wave basis was set as 550 eV. A 10 × 10 × 10 k‐mesh was used to sample the first Brillouin zone. The energies of different magnetic configurations were compared by using the experimental lattice structures.

### Phase Field Simulations

A phase field model was utilized to analyze the effect of dislocations on the twin variant microstructure during the phase transition, in which the total free energy of the system consists of the Landau free energy, gradient energy, elastic energy, and local elastic energy caused by the dislocations.^[^ ^62^ ^]^ The microstructure evolution of the 24 twin variants, according to the Kurdjumov‐Sachs (K‐S) orientation relationship,^[^ ^63^ ^]^ upon demagnetization was governed by the time‐dependent Ginzburg‐Landau equation. The effect of dislocations was described by the elastic interaction between the stress‐free transition strain of martensite variants and the local internal stress fields caused by the dislocations. The details are described in the supplementary information.

## Conflict of Interest

The authors declare no conflict of interest.

## Author Contributions

C.J. and J.W. designed the experiments. Q.Y. performed the sample preparation, phase transition characterizations, and magneto‐superelasticity measurements. J.M. and J.X. performed preferentially oriented martensitic variants characterizations. S.Z. performed dislocation characterizations. C.X. performed measurements at SHMFF. C.L. and D.W. performed phase field simulations. Y.L. and C.S. performed density functional theory calculations. X. X. performed device stroke measurements in the pulsed magnetic field. The manuscript was written by J.W., Q.Y., and C.J. All authors participated in the discussion and analysis of the paper.

## Supporting information

Supporting Information

Movie S1

Movie S2

## Data Availability

Research data are not shared.
