# Supplementary material for: A Giant Magneto‐Superelasticity of 5% Enabled by Introducing Ordered Dislocations in Ni34Co8Cu8Mn36Ga14 Single Crystal
Source: Adv Sci (Weinh). 2024 Apr 24;11(25):2401234. doi: 10.1002/advs.202401234 (PMC11220696; doi:10.1002/advs.202401234)
Supplement: Supplementary file 1 — Supporting Information [file ADVS-11-2401234-s003.pdf]

ADVANCED SCIENCE
Open Access

Supporting Information

for *Adv. Sci.*, DOI 10.1002/advs.202401234

A Giant Magneto-Superelasticity of 5% Enabled by Introducing Ordered Dislocations in $Ni_{34}Co_8Cu_8Mn_{36}Ga_{14}$ Single Crystal

Qijia Yu, Jingmin Wang, Chuanxin Liang, Jiaxi Meng, Jinyue Xu, Yang Liu, Shiteng Zhao, Xuekui Xi, Chuanying Xi, Ming Yang, Chen Si, Yangkun He, Dong Wang and Chengbao Jiang**

Supporting Information for

A giant magneto-superelasticity of 5% enabled by introducing ordered dislocations in $Ni_{34}Co_8Cu_8Mn_{36}Ga_{14}$ single crystal

Qijia Yu, Jingmin Wang, Chuanxin Liang[2], Jiaxi Meng[1], Jinyue Xu[1], Yang Liu[1], Shiteng Zhao[1], Xuekui Xi[3], Chuanying Xi[4], Ming Yang[5], Chen Si[1], Yangkun He[1], Dong Wang[2], Chengbao Jiang[1]**

The PDF file includes:

Supplementary text

Figures S1 to S16

Tables S1 and S2

References

Other supporting information for this manuscript include the following:

Movies S1 and S2

Data S1

Supplementary text

$Ni_{34}Co_8Cu_8Mn_{36}Ga_{14}$ single crystals

Figure S7a shows a rod of a $Ni_{34}Co_8Cu_8Mn_{36}Ga_{14}$ single crystal, with a diameter of 7 mm and a length of 85 mm. Figure S7b shows an optical photograph of the cross section of the $Ni_{34}Co_8Cu_8Mn_{36}Ga_{14}$ single crystal. No grain boundary is observed, confirming the growth of a single crystal. Back-reflection Laue XRD was performed on the cross section of the single crystal at 473 K (the austenite phase) and 300 K (the martensite phase), as shown in Figures S7c and S7d. The single crystal was determined to grow along the $\langle 001 \rangle_A$ direction. The multivariant state of the as-grown single crystal is indicated by various sets of diffraction spots.

Device for SCTC-training

The device is composed of a frame, a screw, and disc springs that are completely made of diamagnetic BeCu alloys, as shown in Figure S8. The magnitude of stress is controlled by the screw through Hook's law.

Thermodynamic analysis of the magnetic field-induced martensitic transformation

For the general temperature-controlled thermoelastic martensitic transformation, the transition thermodynamics are described by the metastable equilibrium temperature T_0 [1,2].

$$\Delta G_{ch}(T_0) = \frac{d\Delta G_C(T_0)}{dT}\Delta T + \delta(\Delta G_{nc}) + \Delta g = 0 \qquad \text{(Equation S1)}$$

where G_{ch} is the chemical free energy, G_{nc} is the nonchemical energy, and Δg is the martensitic transition resistance without elastic strain energy.

The nonchemical energy is defined as the stored elastic strain, that is, the elastic strain energy stored during the martensitic transition and released during the austenitic transition, including the elastic strain energy and interfacial energy [3]. It is defined as

$$\Delta G_{nc} = \Delta G_{el} = \frac{4}{3}\pi r c^2 \mathrm{A} + 2\pi r^2 \sigma \qquad \text{(Equation S2)}$$

where *r* and *c* are the radius and semithickness of the oblate spheroidal martensite particle, respectively. *A* and σ are the elastic strain energy per volume and interfacial energy per area, respectively. ΔG_{el} is the stored elastic energy, which is positive during the austenitic transition and negative during the martensitic transition, resulting in a difference between the starting and finishing temperatures during the transition. The martensitic transition resistance reflects the frictional work, suggesting that there is always a frictional force during the martensitic transition.

For the $Ni_{34}Co_8Cu_8Mn_{36}Ga_{14}$ alloy, the magnetic field-involved energy, including the magnetocrystalline anisotropy energy and Zeeman energy, must be considered.

The magnetocrystalline anisotropy describes the preference for the magnetization to be oriented along certain crystallographic directions. The Zeeman energy describes the potential energy of moments in a field [4]. For strain behavior, the external stress needs to be considered. Therefore, the thermodynamic procedure is described by the equation [5,6]:

$$\Delta G_{total}(T,\sigma,\mu_0 H) = \Delta G_{ch}(T) + \Delta E_{mech}(\sigma) + \Delta G_{el} + \Delta g + \Delta G_{Zeeman}(\mu_0 H) + \Delta G_{MAE}(\mu_0 H) \qquad \text{(Equation S3)}$$

where $\Delta E_{mech}(\sigma)$ is the mechanical energy induced by external stress, $\Delta G_{Zeeman}(\mu_0 H)$ is the Zeeman energy and $\Delta G_{MAE}(\mu_0 H)$ is the magnetocrystalline

anisotropy energy. $\mu_0 H$ is the magnetic field applied. T is the temperature, and σ is the applied stress.

$\Delta G_{ch}(T)$ is described as

$$\Delta G_{ch}(T) = \Delta H - T\Delta S \qquad \text{(Equation S4)}$$

where ΔH is the enthalpy change $H_A - H_M$ and ΔS is the entropy change $S_A - S_M$.

$\Delta E_{mech}(\sigma)$ is described as

$$\Delta E_{mech}(\sigma) = \varepsilon V_m \sigma \qquad \text{(Equation S5)}$$

where ε is the strain change and V_m is the total volume.

$\Delta G_{Zeeman}(\mu_0 H)$ is described as

$$\Delta G_{Zeeman}(\mu_0 H) = \mu_0 H \cdot (M_s^A - M_s^M) \qquad \text{(Equation S6)}$$

where M_s^A is the saturation magnetization of austenite and M_s^M is the saturation magnetization of martensite.

As shown in Figure S9, at temperatures lower than A_s, for example, A_s^H, the driving force simply supplied by $\Delta G_{ch}(T)$ is insufficient for the transition. Fortunately, $\Delta G_{Zeeman}(\mu_0 H)$ supplied by the magnetic field compensates for the additional driving force required at A_s^H. Therefore, the transition can be isothermally induced by the magnetic field at temperatures lower than A_s. However, the required strength of the magnetic field depends on the temperature.

In our system, the applied magnetic field is much greater than the saturation field of the austenite phase, so the magnetocrystalline anisotropy energy can be ignored. The enthalpy, martensitic transition resistance and stored elastic energy barely change with

the temperature and magnetic field. In addition, there is no bias stress applied in our system. Finally, the dependence of the driving magnetic field on temperature is described as follows:

$$\Delta M \cdot d(\mu_0 H) = \Delta S \cdot dT \qquad \text{(Equation S7)}$$

where ΔM is the magnetization change $M_A - M_M$.

When no bias stress is applied, the driving field dependence on temperature is described as follows:

$$\frac{\Delta M}{\Delta S} = \frac{dT}{d(\mu_0 H)} \qquad \text{(Equation S8)}$$

The magnetization change and entropy change related to the phase transition are nearly unaffected by temperature, so the magnetic field required to drive the transition has a linear relationship with temperature.

As mentioned in the main text, the phase transition induced by magnetic field is partially reversible at 310 K. Here, the thermal hysteresis of the phase transition should be considered. As schematically shown in Figure S10, taking the measured *M-T* curves and the phase resulting from the magnetic field, at temperatures in the green area, the magnetic field-induced phase transition is reversible. However, at temperatures in the yellow area, the austenite phase is a stable result of the magnetic field. As a result, at these temperatures, the magnetic field can induce the transition from the martensite phase to the austenite phase, but the austenite phase is retained after removal of the magnetic field. In this case, the magnetic field-induced phase transition is irreversible.

DFT calculations of local atomic moments

For the composition $Ni_{34}Co_8Cu_8Mn_{36}Ga_{14}$, it is difficult to build the lattice structure for DFT calculation. So $Ni_6Co_1Cu_1Mn_6Ga_2$, i.e. $Ni_{37.5}Co_{6.25}Cu_{6.25}Mn_{37.5}Ga_{12.5}$, which is close to the experimental composition $Ni_{34}Co_8Cu_8Mn_{36}Ga_{14}$, is used for DFT calculation. We use the DFT results for qualitative analysis.

In our study, we examined two magnetic configurations for the austenitic phase and three for the martensitic phase, as depicted in Figure S11. The total energies for the various magnetic configurations of the experimental crystal structures were calculated and are presented in Table S1. Our results indicate that the ferromagnetic coupling energy is lower in the austenitic phase (austenite-1), while the antiferromagnetic coupling energy is lower in the martensitic phase (martensite-3). This suggests that all Mn atoms in the austenitic phase are aligned spin-parallel, whereas in the martensite, spins are parallel between nearest-neighbor Mn atoms and opposite between next-nearest-neighbor Mn atoms.

According to the magnetic structure and local magnetic moments calculated for the austenite and martensite phases, as shown in Figure S11, the saturation magnetization of the austenite and martensite phase of the experimental composition $Ni_{34}Co_8Cu_8Mn_{36}Ga_{14}$ is 146 emu/g and 0.2 emu/g, respectively. This indicates that the austenite is a strong ferromagnetic phase and the martensite is a week magnetic phase. This qualitative analysis conclusion is consistent with the experimental results show in Figure S3. So, the results of DFT calculations are reliable and are supported by the experimental magnetic measurement.

Furthermore, we performed total, element-projected, and atom-projected electronic density of states calculations for austenite-1 and martensite-3. The atom labels used in the DFT calculations are shown in Figure S12, and the results of the density of states calculations are presented in Figures S13-S16. We obtained the total and atomic magnetic moments of the structure by integrating the spin-up and spin-down states of the total and atom-projected electronic density of states, respectively.

Phase field model

In the phase field simulation, we consider a martensitic transformation from austenite to martensite with 24 variants according to the K-S orientation relationship [7]. Different phases and different twin variants are represented by the order parameters η_p (p=1, 2, ..., 24) such that the austenite phase is described by $\eta_p(\mathbf{r})=0$ and the pth twin variant is indicated by $\eta_p(\mathbf{r})=1,\ \eta_{q(q\neq p)}(\mathbf{r})=0$. The total free energy F of the model system is described as an integral of the chemical free energy f_{ch}, interfacial energy f_{intf}, elastic energy f_{el} and local elastic energy caused by dislocations f_{local} over the whole system Ω:

$$F=\int_{\Omega}(f_{ch}+f_{intf}+f_{el}+f_{local}) \qquad \text{(Equation S9)}$$

where

$$f_{ch}=\frac{1}{2}A_1\sum_p\eta_p^2(\mathbf{r})-\frac{1}{4}A_2\sum_p\eta_p^4(\mathbf{r})+\frac{1}{6}A_3\left(\sum_p\eta_p^2(\mathbf{r})\right)^3 \qquad \text{(Equation S10)}$$

is the local chemical free energy density approximated by a Landau expansion polynomial, where A_1 is a temperature-dependent coefficient, $A_1=A_1^0[T-T^0]$, and T is the temperature. A_2 and A_3 are constants [8].

The interfacial energy density is described by the nonlocal gradient terms [9]:

$$f_{intf} = \frac{1}{2}\kappa\sum_{p}\left(\nabla\eta_p\right)^2 \quad \text{(Equation S11)}$$

where κ is the interfacial energy coefficient. The elastic energy is calculated with Khachaturyan's microelasticity theory [10]. The local elastic energy density is formulated as [11]

$$f_{local} = -\sum_{p}\sigma_{ij}(\mathbf{r})\cdot\varepsilon_{ij}^{p}\eta_p(\mathbf{r}) \quad \text{(Equation S12)}$$

where $\sigma_{ij}(\mathbf{r})$ is the local stress field caused by the dislocations. ε_{ij}^{p} is the stress-free transformation strain of different twin variants calculated with the deformation gradient tensor $\mathbf{F}_p$:

$$\varepsilon_p = \frac{\mathbf{F}_p^T\mathbf{F}_p - \mathbf{I}}{2} \quad \text{(Equation S13)}$$

where $\mathbf{I}$ is the unit tensor and $\mathbf{F}^T$ is the transpose of $\mathbf{F}$. $\mathbf{F}_p$ can be defined by

$$\mathbf{F}_p = \mathbf{R}_p\mathbf{U} \quad \text{(Equation S14)}$$

where $\mathbf{R}_p$ is the rotation matrix according to the K-S orientation relationship and $\mathbf{U}$ is the Bain stretch tensor [12]:

$$\mathbf{U} = \begin{pmatrix} \sqrt{2}a_M / a_A & 0 & 0 \\ 0 & \sqrt{2}a_M / a_A & 0 \\ 0 & 0 & c_M / a_A \end{pmatrix} \quad \text{(Equation S15)}$$

where a_A=5.85 Å, a_M=3.84 Å and c_M=6.69 Å are the lattice constants of austenite and martensite, which were measured with XRD in our experiments. The time evolution of the order parameters is governed by the stochastic time-dependent Ginsburg-Landau equation:

$$\frac{\partial\eta_p(\mathbf{r},t)}{\partial t} = -L\frac{\delta F}{\delta\eta_p(\mathbf{r},t)} + \xi_p(\mathbf{r},t) \quad \text{(Equation S16)}$$

where L is the kinetic coefficient and ξ is the dimensionless noise term describing thermal fluctuations [13].

In our simulations, the following dimensionless parameters were used: $A_1^0 = 0.05$, $T^0 = 300$, $A_2 = 27.25$, $A_3 = 20.5$, $\kappa = 0.1$, $C_{11} = 152$, $C_{12} = 143$, $C_{44} = 103$, and $M = 1.0$. The length scale of the simulation system was 1 nm.

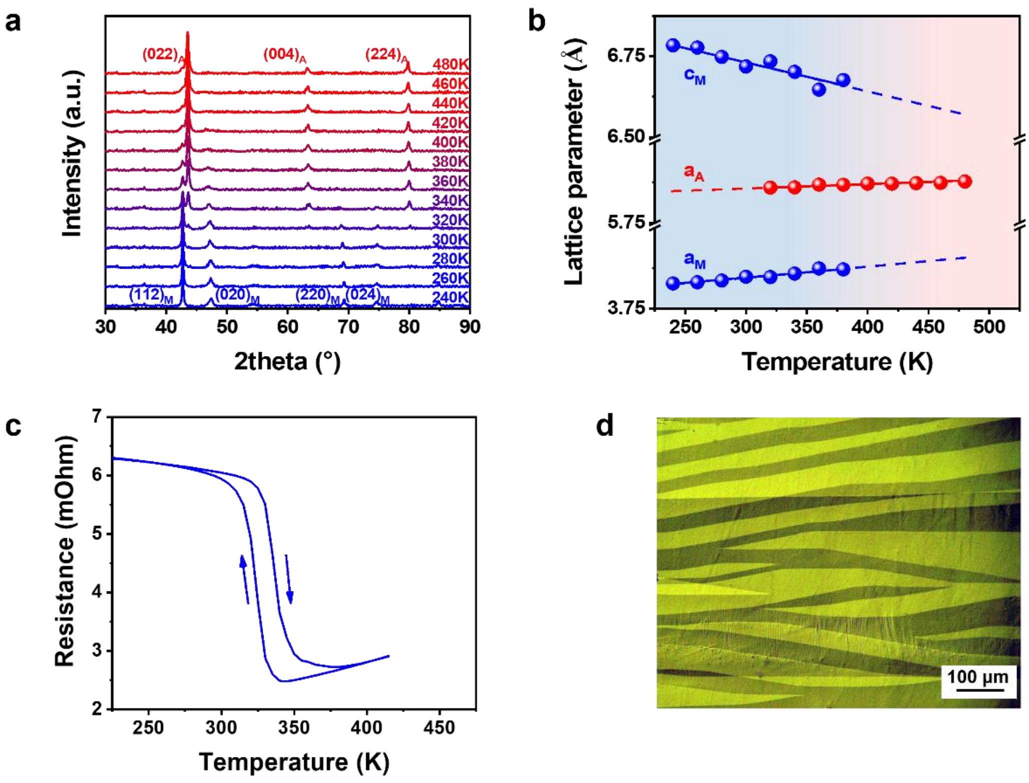

Figure S1. Martensitic transformation of the $Ni_{34}Co_8Cu_8Mn_{36}Ga_{14}$ alloy. a) In situ heating XRD patterns from 240 K to 480 K confirming the structural transition from the bct martensite phase to the fcc austenite phase. b) Temperature dependence of the lattice parameters of martensite and austenite calculated from XRD patterns. c) Temperature dependence of the electrical resistance giving additional evidence of the structural transition. d) Optical photograph of the single crystal observed at 300 K exhibiting a microstructure of randomly oriented martensite variants.

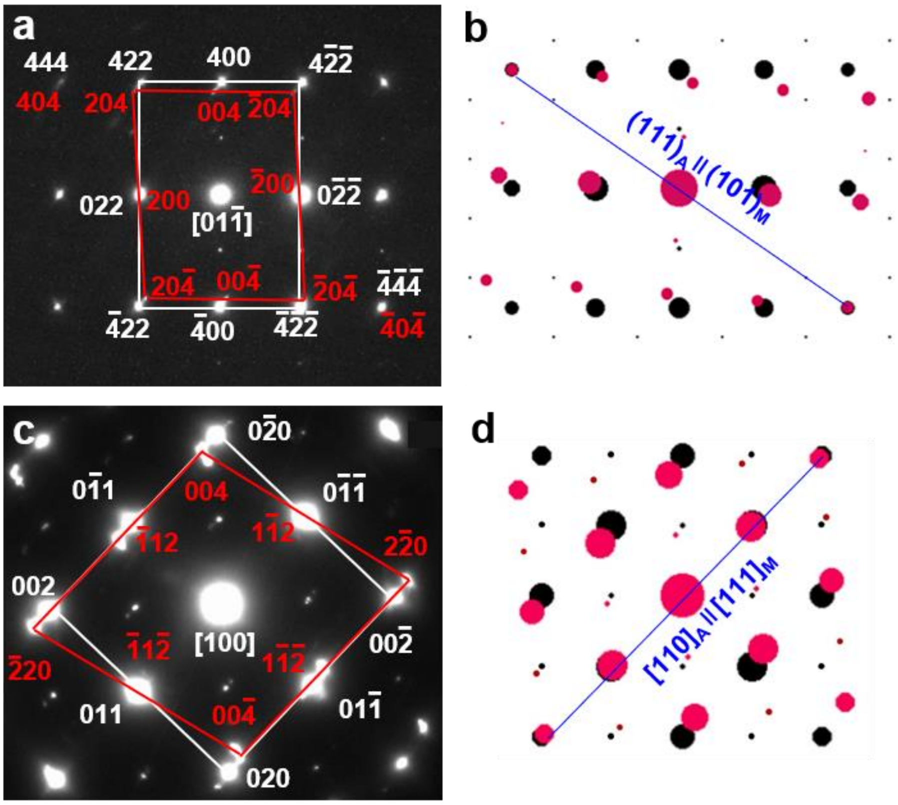

Figure S2. Orientation relationship between the austenite and martensite phases of the $Ni_{34}Co_8Cu_8Mn_{36}Ga_{14}$ alloy. a) and c) SAED patterns of both phases along the $[01\bar{1}]_A$ and $[100]_A$ axes. b) and d) Simulated patterns according to figures a and c, revealing the habit plane and habit direction between the austenite and martensite phases. The red and black spots correspond to diffraction of the martensite and austenite phases, respectively.

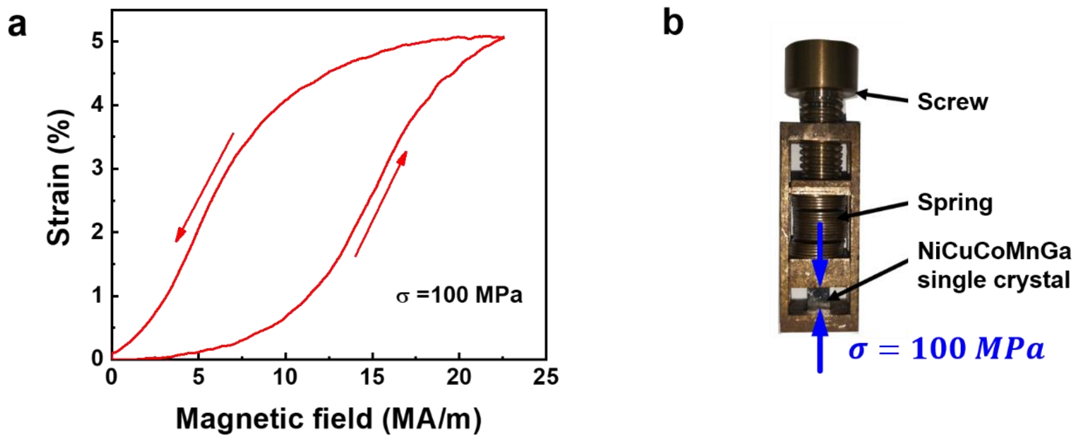

Figure S3. Giant magneto-superelastic strain under compressive stress of 100 Mpa. a) The strain of 5% is still output under the compressive stress of 100 Mpa. b) Device of the $Ni_{34}Co_8Cu_8Mn_{36}Ga_{14}$ single crystal under the compressive stress.

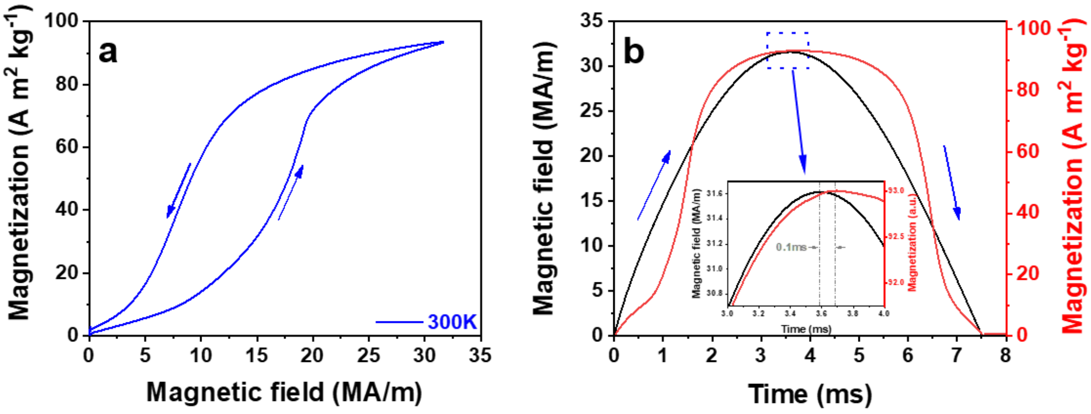

Figure S4. Magnetization under pulsed magnetic field of the $Ni_{34}Co_8Cu_8Mn_{36}Ga_{14}$ alloy. a) Magnetic field dependence of magnetization measured at 300 K in a pulsed field with the frequency width of 8 ms. b) Time dependence of the pulsed magnetic field and the corresponding magnetization evidencing that the fast response of the magnetostructural transition to the field.

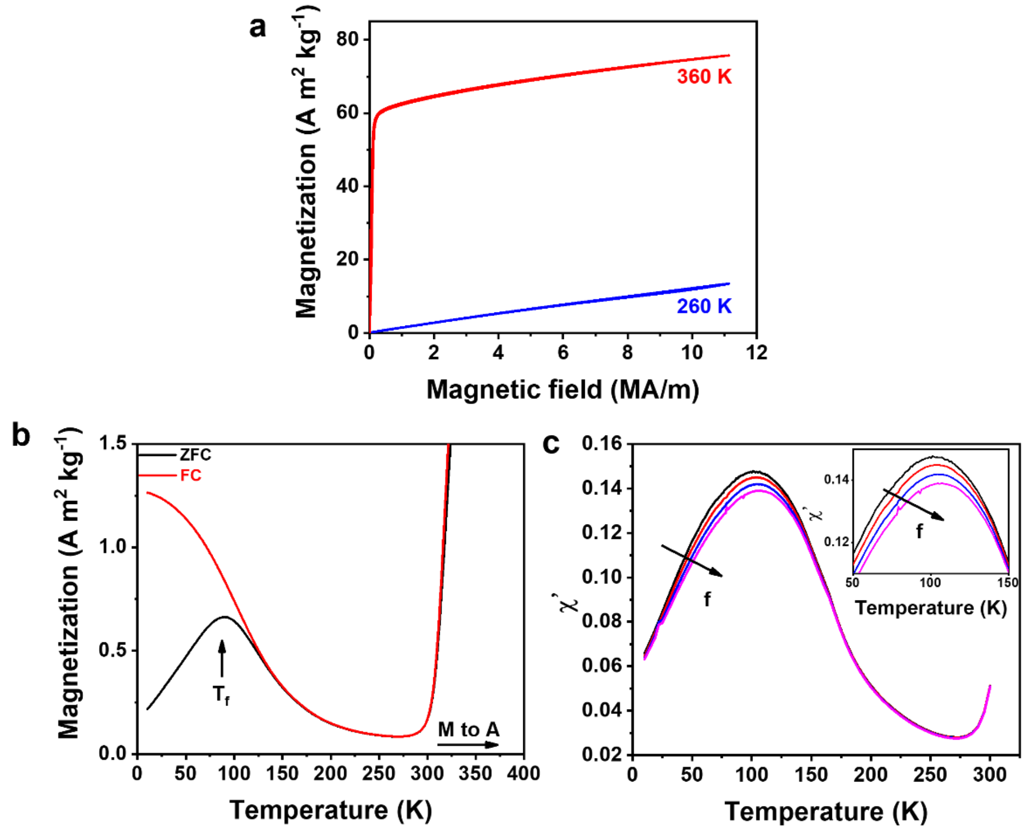

Figure S5. Magnetism analysis of the $Ni_{34}Co_8Cu_8Mn_{36}Ga_{14}$ alloy. a) Magnetization curves measured for the austenite phase at 360 K and for the martensite phase at 260 K. b) Temperature dependence of the magnetization measured under ZFC/FC conditions, where T_f means the freezing temperature of the spin glass. c) Temperature dependence of the AC susceptibility measured with frequencies of 5 Hz, 19 Hz, 71 Hz, 266 Hz and 1000 Hz and a field amplitude of 3 Oe. The inset shows a partial enlargement of the curves.

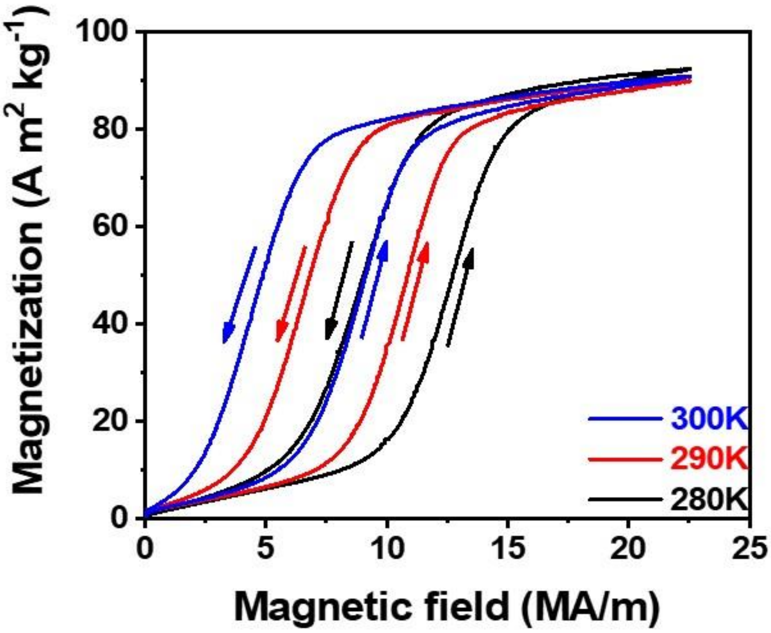

Figure S6. Magnetic field-induced martensitic transformation. Dependences of magnetization are measured at temperatures of 280 K, 290 K and 300 K. The abrupt change of the magnetization and the obvious hysteresis of the curves, together with the structural analysis (Figure S2, Supporting Information), give evidence of the reversible martensitic transformation induced by the magnetic field.

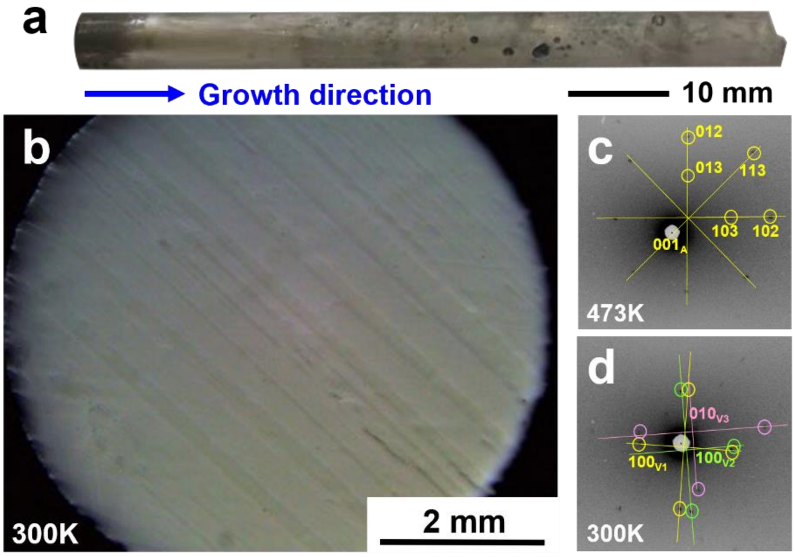

Figure S7. $Ni_{34}Co_8Cu_8Mn_{36}Ga_{14}$ single crystal. a) Image of the as-grown single crystal rod. b) Optical photograph of the cross-section of the single crystal. c) and d) Back-reflection Laue XRD patterns detected at 473 K and 300 K.

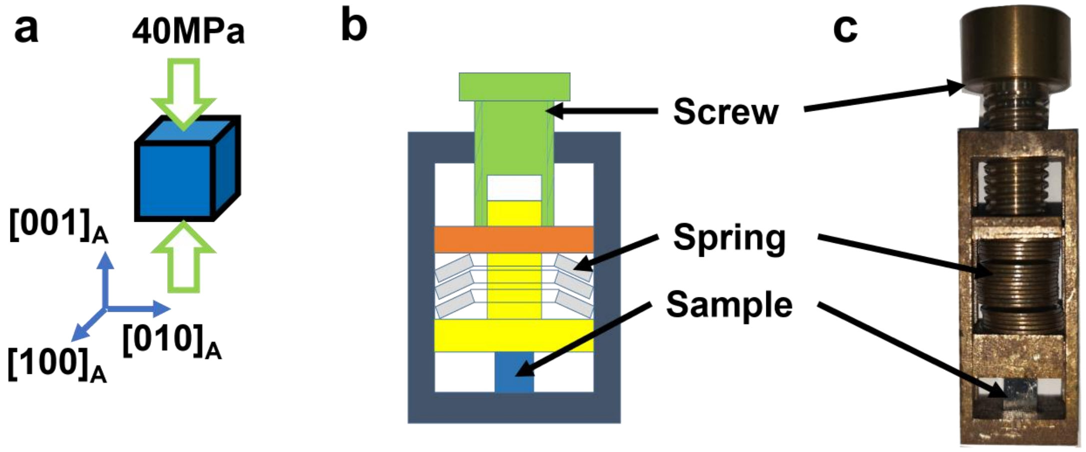

Figure S8. Device for SCTC training. a) Schematic illustration of applying compressive stress on the single crystal. b) and c) Structure of the device.

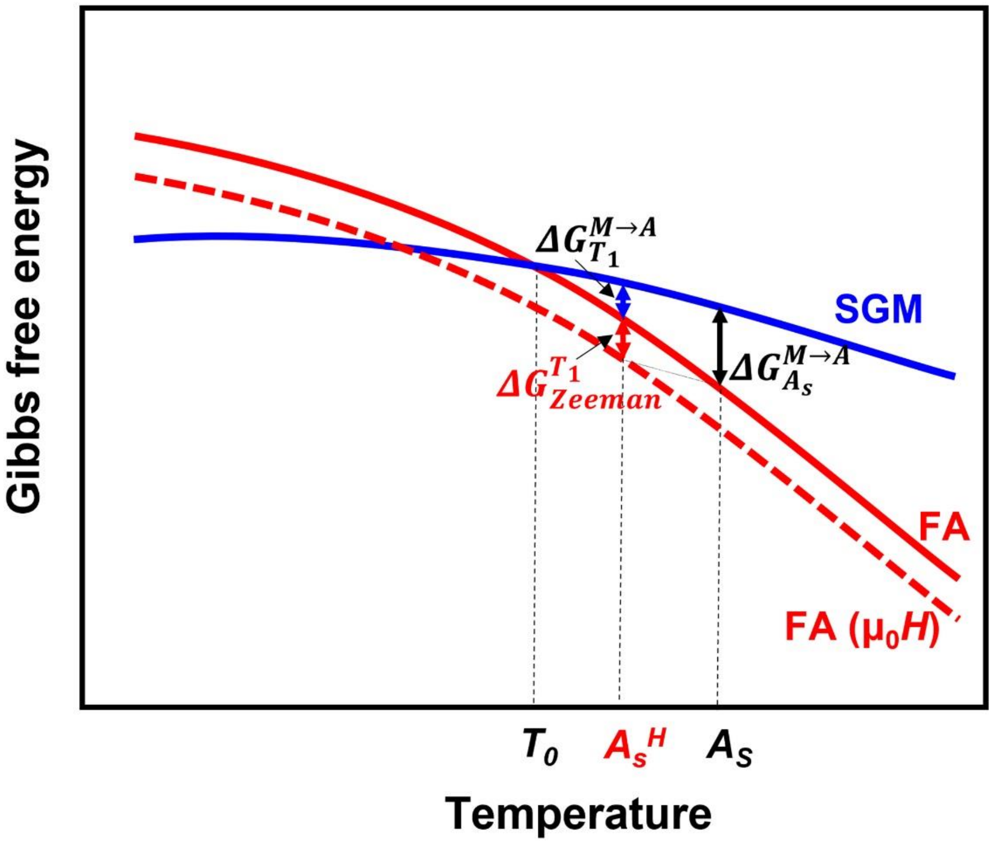

Figure S9. Thermodynamic analysis of the magnetic field-induced martensitic transformation.

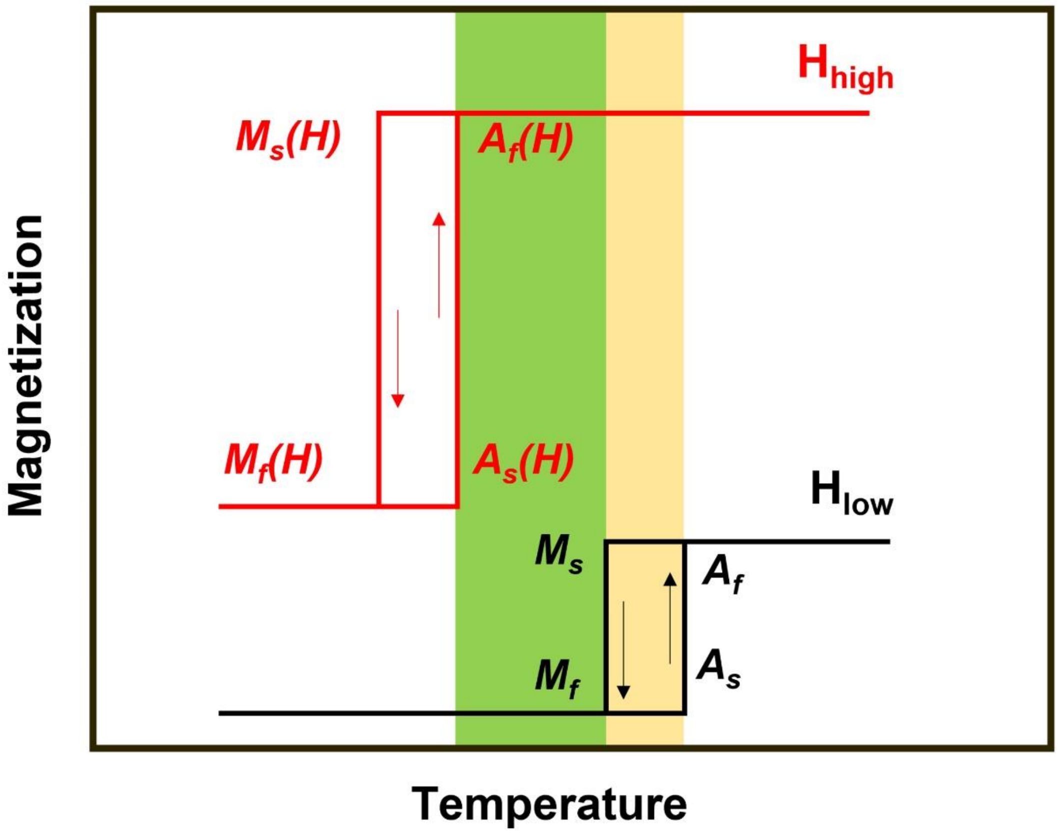

Figure S10. Schematic illustration of the temperature ranges of reversible or irreversible martensitic transformation under a magnetic field.

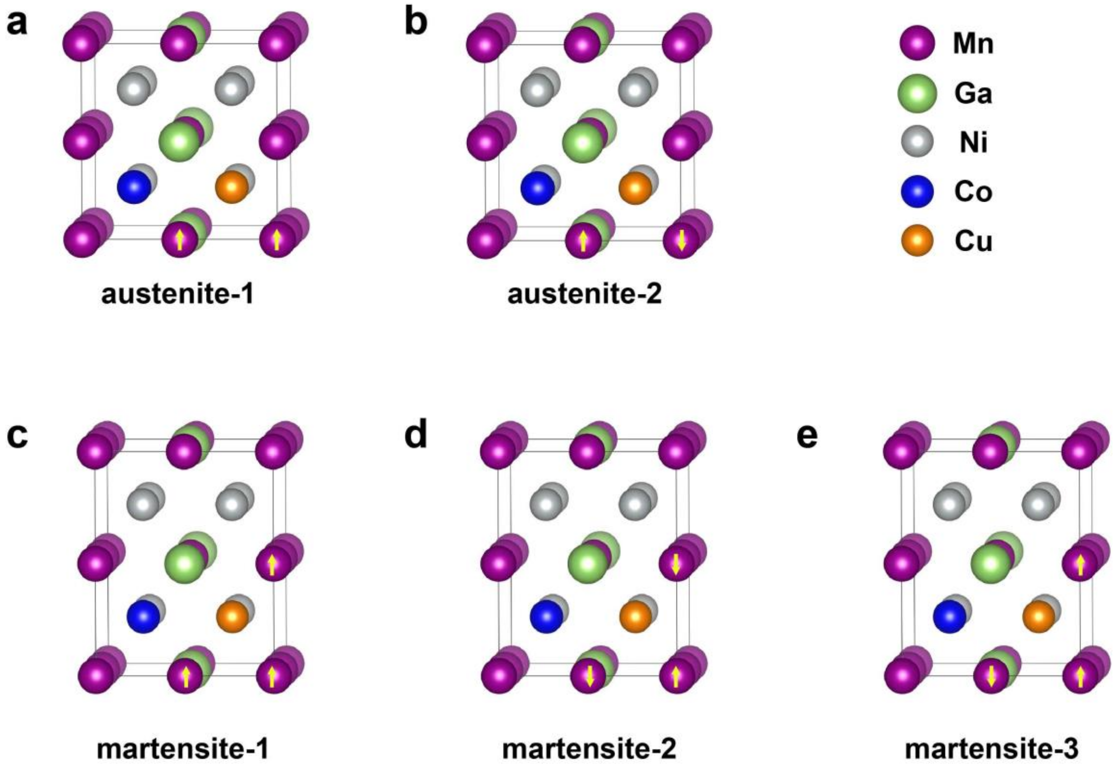

Figure S11. Magnetic configurations used for DFT calculations in the $Ni_6Cu_1Co_1Mn_6Ga_2$ unit cell. a) Ferromagnetic coupling of all Mn atoms in austenite. b) Antiferromagnetic coupling of nearest-neighbor Mn atoms and ferromagnetic coupling of next-nearest-neighbor Mn atoms in austenite. c) Ferromagnetic coupling of all Mn atoms in martensite. d) and e) Antiferromagnetic coupling of nearest-neighbor Mn atoms along the *a*-axis, while the nearest-neighbor Mn atoms along the *c*-axis are antiferromagnetically d) and ferromagnetically e) coupled.

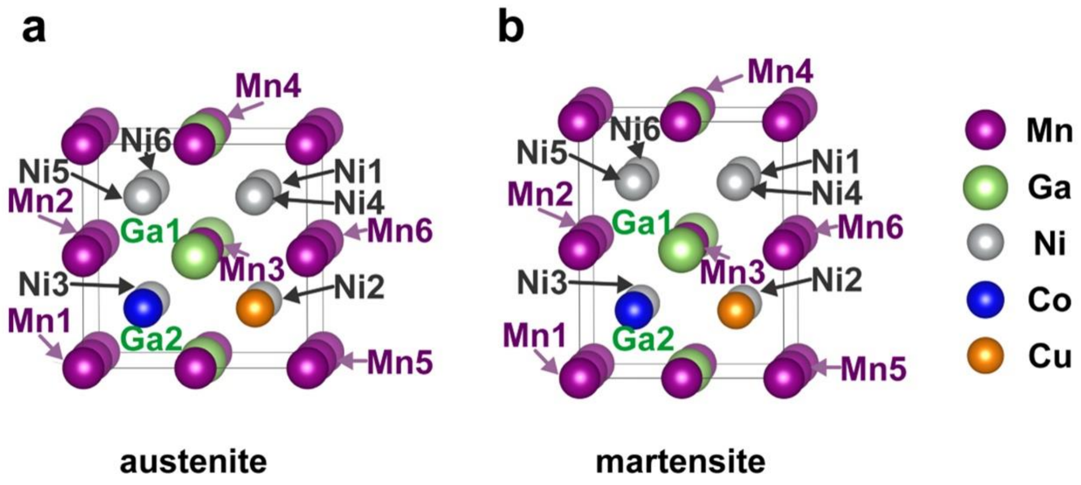

Figure S12. All atoms are labeled in the $Ni_6Cu_1Co_1Mn_6Ga_2$ unit cell for DFT calculations.

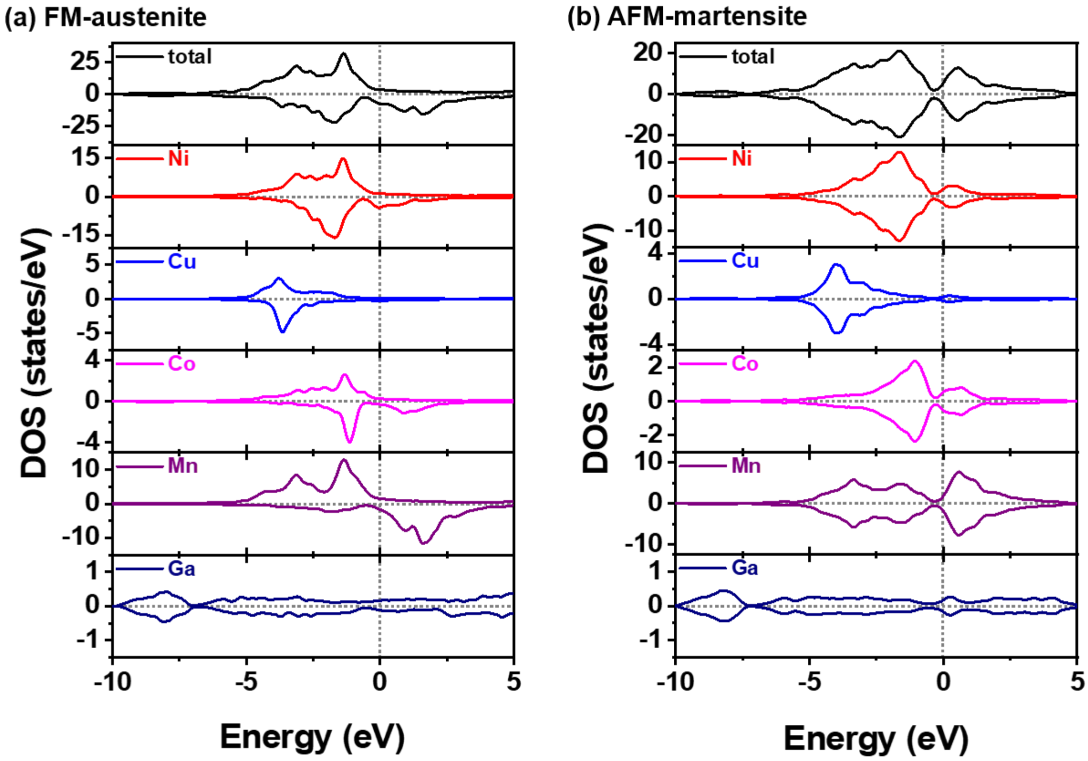

Figure S13. Total and element-projected density of states (DOS) in the $Ni_6Cu_1Co_1Mn_6Ga_2$ unit cell. a) Ferromagnetic austenite phase. b) Antiferromagnetic martensite phase. Spin-up and spin-down DOSs are shown as positive and negative y-values. The x-axis is shifted to bring the Fermi level to 0 eV.

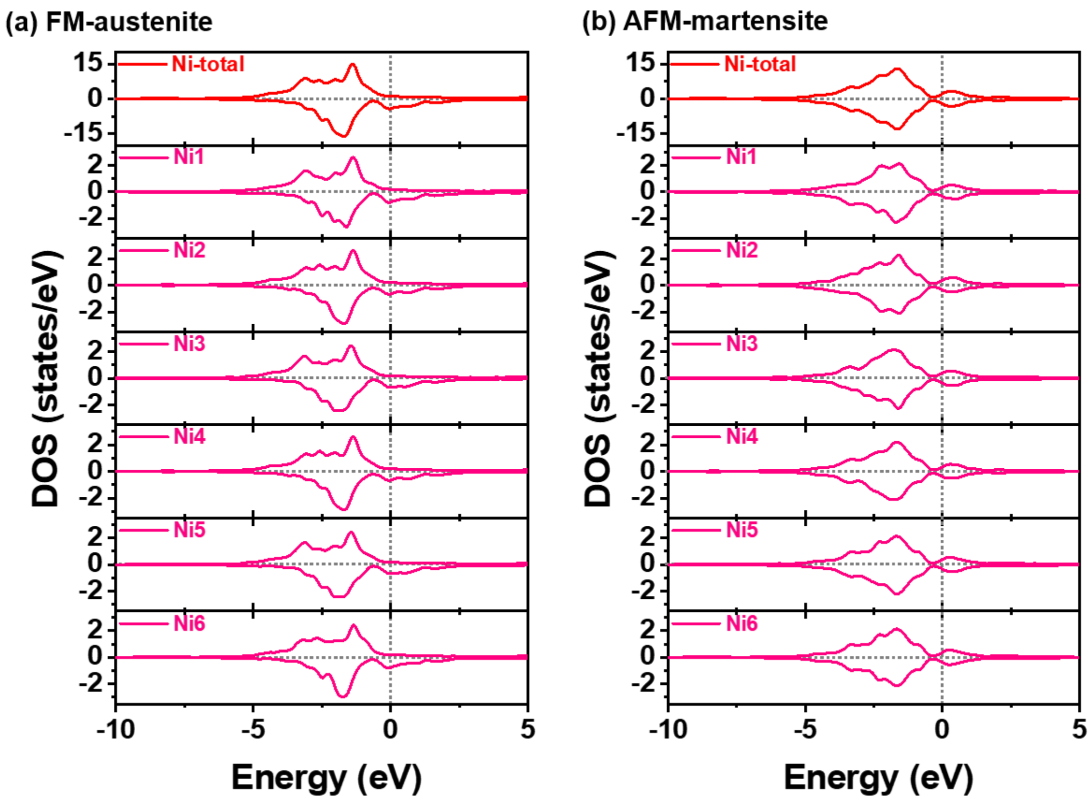

Figure S14. Atom-projected density of states (DOS) of Ni in the $Ni_6Cu_1Co_1Mn_6Ga_2$ unit cell. a) Ferromagnetic austenite phase. b) Antiferromagnetic martensite phase. Spin-up and spin-down DOSs are shown as positive and negative y-values. The x-axis is shifted to bring the Fermi level to 0 eV.

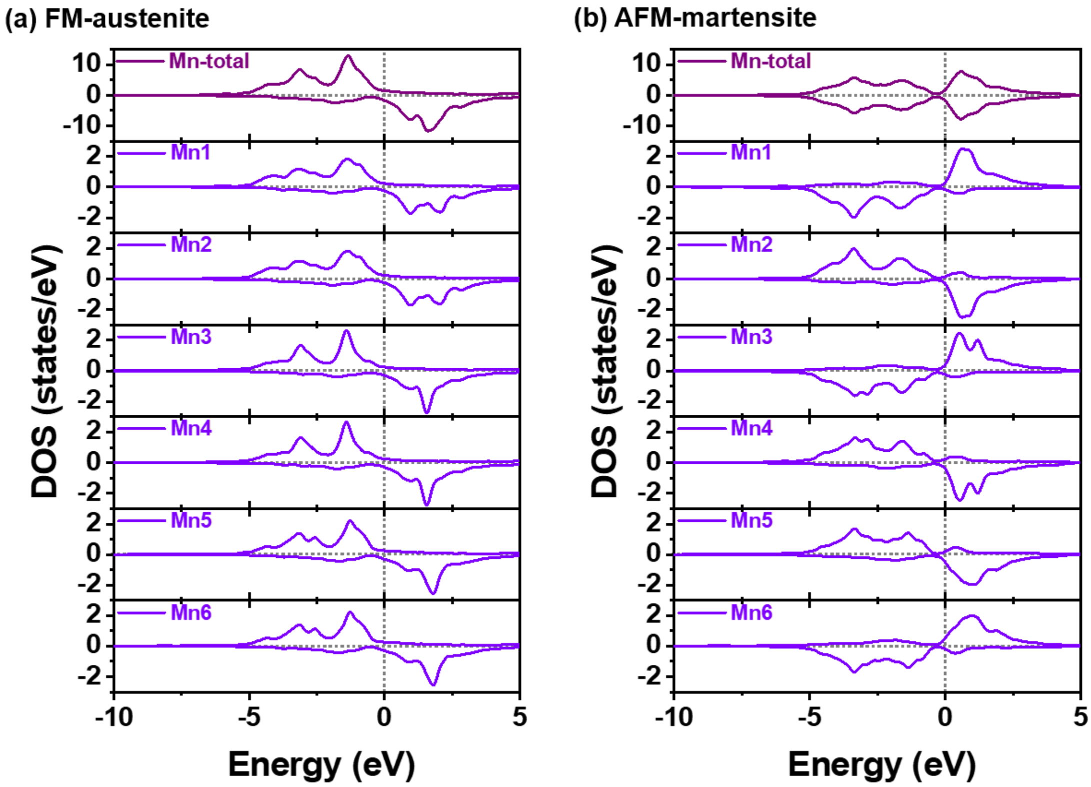

Figure S15. Atom-projected density of states (DOS) of Mn in the $Ni_6Cu_1Co_1Mn_6Ga_2$ unit cell. a) Ferromagnetic autensite phase. b) Antiferromagnetic martensite phase. Spin-up and spin-down DOSs are shown as positive and negative y-values. The x-axis is shifted to bring the Fermi level to 0 eV.

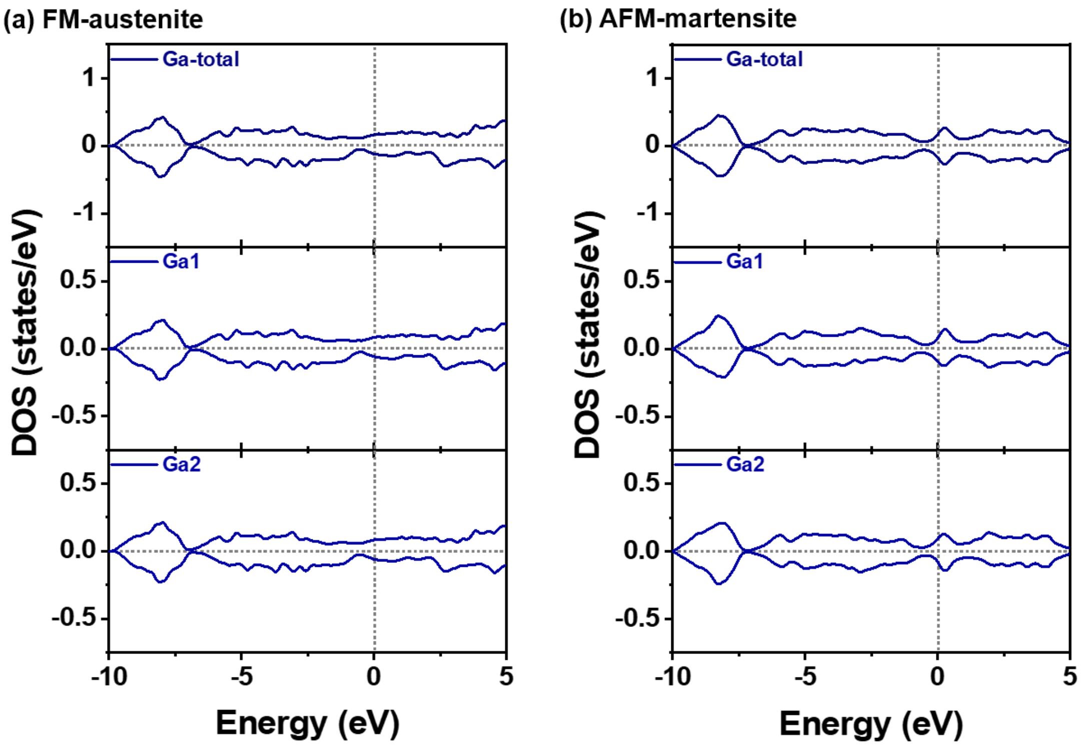

Figure S16. Atom-projected density of states (DOS) of Ga in the $Ni_6Cu_1Co_1Mn_6Ga_2$ unit cell. a) Ferromagnetic austenite phase. b) Antiferromagnetic martensite phase. Spin-up and spin-down DOSs are shown as positive and negative y-values. The x-axis is shifted to bring the Fermi level to 0 eV.

Table S1. Total energy and total moments of austenite and martensite in Figure S11.

Structure	Total energy/eV	Total moments/μ_B
austenite-1	-105.116	25.60
austenite-2	-104.706	0.67
martensite-1	-104.969	25.02
martensite-2	-105.377	8.27
martensite-3	-105.463	-0.04

Table S2. Local atomic moments in the austenite and martensite phases of the NiCuCoMnGa alloy. Ni1, Ni2, Ni3, etc are different atom of occupations described in the supplementary information Figure S12.

moment / μ_B	Ni1	Ni2	Ni3	Ni4	Ni5	Ni6	Co1	Cu1
austenite	0.58	0.56	0.57	0.54	0.66	0.56	1.37	0.10
martensite	0	0	0	0	0	0	-0.03	0
**moment / μ_B**	**Mn1**	**Mn2**	**Mn3**	**Mn4**	**Mn5**	**Mn6**	**Ga1**	**Ga2**
austenite	3.52	3.52	3.47	3.47	3.41	3.40	-0.06	-0.06
martensite	-3.42	3.42	-3.39	3.39	3.31	-3.31	0.05	-0.05

Movie S1

Large stroke is resulted from the giant magneto-superelasticity.

Movie S2

The stroke is rather small in case of the small magneto-elastic strain.

Data S1

The martensitic transition strain matrix from the austenite phase to the martensite phase.

Variant 1: $\begin{bmatrix} 0.036018 & -0.112586 & -0.004601 \\ -0.112586 & 0.052968 & 0.00496 \\ -0.004601 & 0.00496 & -0.068209 \end{bmatrix}$

Variant 2: $\begin{bmatrix} -0.044391 & -0.053997 & 0.044004 \\ -0.053997 & 0.052968 & -0.098917 \\ 0.044004 & -0.098917 & 0.0122 \end{bmatrix}$

Variant 3: $\begin{bmatrix} -0.050753 & -0.042846 & 0.042936 \\ -0.042846 & 0.035546 & -0.104177 \\ 0.042936 & -0.104177 & 0.035984 \end{bmatrix}$

Variant 4: $\begin{bmatrix} -0.06603 & -0.001267 & 0.023044 \\ -0.001267 & -0.067737 & -0.012263 \\ 0.023044 & -0.012263 & 0.154545 \end{bmatrix}$

Variant 5: $\begin{bmatrix} -0.050753 & -0.042846 & 0.042936 \\ -0.042846 & 0.035546 & -0.104177 \\ 0.042936 & -0.104177 & 0.035984 \end{bmatrix}$

Variant 6: $\begin{bmatrix} -0.06603 & -0.001267 & 0.023044 \\ -0.001267 & -0.067737 & -0.012263 \\ 0.023044 & -0.012263 & 0.154545 \end{bmatrix}$

Variant 7: $\begin{bmatrix} -0.06603 & 0.001267 & 0.023044 \\ 0.001267 & -0.067737 & 0.012263 \\ 0.023044 & 0.012263 & 0.154545 \end{bmatrix}$

Variant 8: $\begin{bmatrix} -0.050753 & 0.042846 & 0.042936 \\ 0.042846 & 0.035546 & 0.104177 \\ 0.042936 & 0.104177 & 0.035984 \end{bmatrix}$

Variant 9: $\begin{bmatrix} -0.044391 & 0.053997 & 0.044004 \\ 0.053997 & 0.052968 & 0.098917 \\ 0.044004 & 0.098917 & 0.0122 \end{bmatrix}$

Variant 10: $\begin{bmatrix} 0.036018 & 0.112586 & -0.004601 \\ 0.112586 & 0.052968 & -0.00496 \\ -0.004601 & -0.00496 & -0.068209 \end{bmatrix}$

Variant 11: $\begin{bmatrix} -0.050753 & -0.042846 & 0.042936 \\ -0.042846 & 0.035546 & -0.104177 \\ 0.042936 & -0.104177 & 0.035984 \end{bmatrix}$

Variant 12: $\begin{bmatrix} -0.06603 & -0.001267 & 0.023044 \\ -0.001267 & -0.067737 & -0.012263 \\ 0.023044 & -0.012263 & 0.154545 \end{bmatrix}$

Variant 13: $$\begin{bmatrix} 0.050789 & 0.111319 & -0.018443 \\ 0.111319 & 0.035546 & -0.017223 \\ -0.018443 & -0.017223 & -0.065558 \end{bmatrix}$$

Variant 14: $$\begin{bmatrix} 0.115921 & 0.011151 & -0.08694 \\ 0.011151 & -0.067737 & -0.005259 \\ -0.08694 & -0.005259 & -0.027407 \end{bmatrix}$$

Variant 15: $$\begin{bmatrix} -0.06603 & 0.001267 & 0.023044 \\ 0.001267 & -0.067737 & 0.012263 \\ 0.023044 & 0.012263 & 0.154545 \end{bmatrix}$$

Variant 16: $$\begin{bmatrix} -0.050753 & 0.042846 & 0.042936 \\ 0.042846 & 0.035546 & 0.104177 \\ 0.042936 & 0.104177 & 0.035984 \end{bmatrix}$$

Variant 17: $$\begin{bmatrix} -0.044391 & 0.053997 & 0.044004 \\ 0.053997 & 0.052968 & 0.098917 \\ 0.044004 & 0.098917 & 0.0122 \end{bmatrix}$$

Variant 18: $$\begin{bmatrix} 0.036018 & 0.112586 & -0.004601 \\ 0.112586 & 0.052968 & -0.00496 \\ -0.004601 & -0.00496 & -0.068209 \end{bmatrix}$$

Variant 19: $$\begin{bmatrix} 0.036018 & -0.112586 & -0.004601 \\ -0.112586 & 0.052968 & 0.00496 \\ -0.004601 & 0.00496 & -0.068209 \end{bmatrix}$$

Variant 20: $$\begin{bmatrix} -0.044391 & -0.053997 & 0.044004 \\ -0.053997 & 0.052968 & -0.098917 \\ 0.044004 & -0.098917 & 0.0122 \end{bmatrix}$$

Variant 21: $$\begin{bmatrix} -0.050753 & -0.042846 & 0.042936 \\ -0.042846 & 0.035546 & -0.104177 \\ 0.042936 & -0.104177 & 0.035984 \end{bmatrix}$$

Variant 22: $$\begin{bmatrix} -0.06603 & -0.001267 & 0.023044 \\ -0.001267 & -0.067737 & -0.012263 \\ 0.023044 & -0.012263 & 0.154545 \end{bmatrix}$$

Variant 23: $$\begin{bmatrix} 0.115921 & -0.011151 & -0.08694 \\ -0.011151 & -0.067737 & 0.005259 \\ -0.08694 & 0.005259 & -0.027407 \end{bmatrix}$$

Variant 24: $$\begin{bmatrix} 0.050789 & -0.111319 & -0.018443 \\ -0.111319 & 0.035546 & 0.017223 \\ -0.018443 & 0.017223 & -0.065558 \end{bmatrix}$$

References

[1] H. C. Tong, C. M. Wayman, *Scripta Mater.* **1974**, *8*, 93-100.

[2] H. C. Tong, C. M. Wayman, *Acta Metall.* **1974**, *22*, 887-896.

[3] P. Wollants, J. R. Roos, L. Delaey, *Prog. Mater. Sci.* **1993**, *37*, 227-288.

[4] R. C. Chandley, *Modern and magnetic materials: principles and applications*. A Wiley-Interscience publication. ch. 8, **1999**.

[5] H. E. Karaca, I. Karaman, B. Basaran, D.C. Lagoudas,Y.I. Chumlyakov, H.J. Maier *Acta Mater*. **2007**, *55*, 4253-4269.

[6] H. E. Karaca, I. Karaman, B. Basaran, Y. Ren, Y. I. Chumlyakov, H. J. Maier, *Adv. Funct. Mater.* **2009**, *19,* 983-998.

[7] K. Koumatos, A. A Muehlemann, *Acta Cryst. A*. **2017**, *73*. 115-123.

[8] D. Wang, S. Hou, Y, Wang, X. D. Ding, S. Ren, X. B. Ren, Y. Z. Wang, *Acta Mater*. **2014**, *66*, 349-359.

[9] J. W. Cahn, *Acta Metall.* **1961**, *9*, 795-801.

[10] A. G. Khachaturyan, *Theory of structural transformations in solids*. Courier Corporation, **2013**.

[11] C. X. Liang, D. Wang, Z. Wang, X. D. Ding, Y. Z. Wang, *Acta Mater. 194*, **2020**, 134-143.

[12] O. Shchyglo, G. Du, J. K. Engels, I. Steinbach, *Acta Mater*. **2019**, *175.* 415-425.

[13] K Elder, H. Gould, J. Tobochnik, *Comput. Phys.* **1993**, *7,* 27.
